# Detection of pathogenic *Leptospira* with rapid extraction followed by recombinase polymerase amplification (RPA) and quantitative polymerase chain reaction (qPCR) assay-A comprehensive study from Sri Lanka

**DOI:** 10.1371/journal.pone.0295287

**Published:** 2024-03-15

**Authors:** Hansi Uduwawala, Aresha Manamperi, Gayana P. S. Gunaratna, Lilani Karunanayake, Arianna Ceruti, Ahmed Abd El Wahed, Lakkumar Fernando, Ranjan Premaratna, Menaka Hapugoda

**Affiliations:** 1 Molecular Medicine Unit, Faculty of Medicine, University of Kelaniya, Ragama, Sri Lanka; 2 Department of Medical Microbiology, Faculty of Medicine, University of Kelaniya, Ragama, Sri Lanka; 3 National Reference Laboratory for Leptospirosis, Medical Research Institute, Colombo, Sri Lanka; 4 Institute of Animal Hygiene and Veterinary Public Health, Leipzig University, Leipzig, Germany; 5 Centre for Clinical Management of Dengue & Dengue Haemorrhagic Fever, District General Hospital, Negombo, Sri Lanka; 6 Department of Medicine, Faculty of Medicine, University of Kelaniya, Ragama, Sri Lanka; University of Kentucky College of Medicine, UNITED STATES

## Abstract

Leptospirosis is the most widespread zoonosis in the world. The disease is more prevalent in tropical regions where the majority of developing countries are located. Leptospirosis is considered a protean manifestation zoonosis with severity of the disease ranging from a mild febrile illness to a severe and life-threatening illness. Clinical symptoms of leptospirosis overlap with other tropical febrile illnesses. Early, rapid, and definitive diagnosis is important for effective patient management. Since Polymerase Chain Reaction (PCR)-based assays are not readily available in most clinical settings, there is a need for an affordable, simple, and rapid diagnostic test. Quantitative PCR (qPCR) and Recombinase Polymerase Amplification (RPA) were implemented at the Faculty of Medicine, University of Kelaniya, and a prospective study to evaluate RPA for diagnosis of acute phase of leptospirosis was conducted. Results indicate that RPA and qPCR were positive in 81% (98/121) of the total positive and acute clinical samples. Of the 81 positive MAT confirmed patients 60 (74%) and 53 (65%) were positive with qPCR and RPA respectively. Retrospective evaluation revealed a high diagnostic accuracy (sensitivity-70% and specificity-87%) of RPA compared to MAT as the reference gold standard. Results further suggest that there is no significant difference between the two assays, qPCR and RPA-SwiftX (P = 0.40). Laboratory procedures for the extraction and detection by qPCR in the laboratory have been optimized to obtain results within 6 hours. However, the RPA-SwiftX method under field conditions took 35 minutes. The RPA-SwiftX method could replace the qPCR which shows similar sensitivity and specificity. Therefore, RPA established under the current study presents a powerful tool for the early and rapid diagnosis of leptospirosis at point-of-care.

## Introduction

Leptospirosis is an acute febrile illness caused by a pathogenic spirochete of the genus *Leptospira* [1]. The disease is one of the most widely spread zoonosis in the world with an incidence of 1·03 million and 58,900 deaths reported annually [2]. Rodents are considered the most important and widely disseminated reservoirs for this zoonosis, although farm animals and livestock can also harbor the infection. Humans acquire the infection through direct contact with the urine of infected or reservoir animals or following indirect exposure to environments contaminated with urine carrying the organism [1, 3]. Leptospirosis has been classified as a neglected emerging infectious disease due to acquisition of the organism by occupational (veterinarians, farmers, abattoir workers, and rodent control workers) or recreational activities and exposure during heavy rainfall and flooding [1, 4]. Indirect contact with contaminated wet soil or water is considered responsible for the great majority of cases in tropical countries including in Sri Lanka. This is either through occupational exposure such as in farming, with flooding after heavy rains, or exposure to damp soil and water during avocational activities. However, contamination due to recreational exposures is increasing, often in association with adventure eco-tourism in tropical endemic areas [5].

Patients with leptospirosis present to acute care settings with mild to severe life-threating disease. The common clinical manifestations of mild disease include fever, myalgia, malaise, conjunctivitis, anorexia, nausea, vomiting, and abdominal pain. Patients with severe disease present with evidence of single or multiple organ involvement. The initial signs of severe disease include jaundice, hemorrhage, oliguria, shortness of breath, and hepatosplenomegaly [1, 4, 6]. The most severe form of leptospirosis manifests as Weil syndrome with hepatic and renal failure or with massive pulmonary hemorrhages. However, the diagnosis of leptospirosis is challenging even in disease-prevalent settings due to highly variable nonspecific clinical presentations. Moreover, the disease manifestations mimic other tropical infectious diseases such as dengue fever, hantavirus infection, hepatitis, typhoid, and malaria, further challenging medical care providers [7, 8].

Early diagnosis and timely commencement of antibiotics and other supportive care are considered essential for management of patients with leptospirosis to obtain a favorable outcome. The usual practice in most settings is to diagnose the disease clinically and to obtain laboratory confirmation retrospectively. The Microscopic Agglutination Test (MAT) is the most widely used laboratory test for the retrospective confirmation of the clinical diagnosis of leptospirosis [9, 10]. The test detects anti-*Leptospira* antibodies at the late acute phase (after 5 to 7 days) of the disease [1, 11]. Therefore, patients suspected of leptospirosis are likely to receive unnecessary broad-spectrum antibiotics and undergo extensive investigations. The availability of a reliable acute phase diagnostic test is likely to prevent such inappropriate care and to reduce morbidity and mortality associated with the illness. It would curtail overall healthcare costs.

*Leptospira* is a slow-growing bacterium that takes weeks to become positive in culture-based methods and thus, is not clinically beneficial in acute management of patients [12, 13]. On the other hand, the acute phase diagnosis can be obtained by conducting Polymerase Chain Reaction (PCR)-based diagnostic tests with high sensitivity and specificity [11, 14]. However, for disease-endemic low-income countries, the establishment of such facilities is challenging due to the high cost and need for sophisticated laboratory facilities. Moreover, PCR-based molecular tests carry a reasonably high turnaround time [15]. Therefore, the establishment of low cost, simple, and rapid diagnostic test with high sensitivity and specificity is essential. The molecular-based isothermal applications assays have been developed as affordable, simple alternatives for diagnosis of other infectious diseases and are in wide use [15]. The Recombinase Polymerase Amplification (RPA) is an isothermal technique developed to detect leptospirosis in the acute phase of the infection. The ability to use the technique in point of care diagnosis is an additional benefit, especially in low resource settings [15, 16]. Therefore, this prospective study aimed to evaluate the RPA assay and rapid extraction, for the acute phase diagnosis of leptospirosis.

## Materials and methods

A prospective study was conducted with a consecutive cohort of 140 patients clinically suspected of having leptospirosis and admitted for inpatient care to 4 main government hospitals (Colombo North Teaching Hospital, District General Hospital Gampaha and Negombo, and Base Hospital Wathupitiwala) in the leptospirosis endemic Gampaha district, Sri Lanka. Clinical diagnosis of leptospirosis was based on the surveillance case definition published by the Epidemiology Unit, Ministry of Health, Sri Lanka [17]. The study was carried out over a period of 17 months from 1st of December 2018 to 1^st^ of April, 2020. The index tests qPCR and RPA were performed prior to obtaining MAT results.

### Data collection

According to the Inclusion criteria, the patients aged 12 years or older, had a history of fever with a duration of 10 days or less and had leptospirosis as one of the possible clinical diagnoses recorded by the admitting physician were included in the study.

Patients with any other confirmed diagnosis for the febrile illness, inability to provide clinical information at the sample collection, and refusal to participate in the study were excluded. Index or reference test with missing/misplaced data will be excluded from the study (S1 Fig). All the samples were prospectively included in the study. Basic demographic and clinical data were obtained using a pretested interviewer-administered questionnaire including a data collection form with a participant information sheet. All the selected participants or relatives, if the patient were unconscious or aged less than 18 years were eligible for the study with informed written consent. All the relevant data from clinical and other investigations were obtained using hospital records (bed head ticket).

### Ethical considerations

The study protocol was approved by the Ethical Review Committee of the Faculty of Medicine, University of Kelaniya (Reference no: P/207/08/2017).

### Clinical specimen collection

Blood samples were collected into EDTA tubes from patients suspected of having leptospirosis with 1 to 7 days of onset of disease. Five milliliters (5 ml) of convalescent blood sample was collected from the same patient after 7–10 days of DPO for the MAT in a plain tube. All collected blood samples were centrifuged at 6,500 rpm for 10 minutes to separate serum and plasma and stored at -20°C for further testing.

### Laboratory confirmation by RPA-SwiftX assay

#### Rapid extraction using SwiftX

Total DNA from serum was extracted using a commercial kit SwiftX (Xpedite, Munich, Germany) with an additional enrichment step for blood according to the manufacturer’s instructions. The nucleic acid extraction protocol of SwiftX was performed with enrichment step as follows: 1,000 μL of enrichment buffer and 30 μL of magnetic beads were added to the 500 μL of serum samples. The mixture was vortexed for 10s and incubated at room temperature (~28°C) for 3 minutes. Supernatant was discarded after 1 minute incubation in the magnetic rack. Five hundred microliters (500 μL) of lysis buffer (buffer SL) was added to the remaining magnetic beads and vortexed for 10s. Subsequently, sample was incubated at room temperature (~28°C) for 3 minutes and supernatant discarded after 1 minute incubation in the magnetic rack. One hundred (100 μL) of Lysis buffer (buffer SL) was added to the sample and incubated at 95°C. Every 2 minutes, the tube was taken out from the heat block and vortexed. Following 15 minutes of incubation time, the tube was placed on a magnetic rack. After 2 minutes, 50 μL of the supernatant was pipetted out for further testing. Each extraction set included nuclease-free distilled water (500 μL) as the extraction negative control to monitor the cross-contamination during the extraction phase.

#### RPA reagents and reaction conditions

The RPA assay was performed according to the optimized assay reaction conditions and reagents used by Ahmed *et al*. (2014) for the detection of pathogenic *Leptospira* [15]. TwistAmp exo probe lyophilized kit (TwistDx, Ltd., Cambridge, UK) was supplied as vacuum-sealed strips of eight reactions in ready- to-use tubes to perform the RPA assay as follows: 29.5 μl of the rehydration buffer, 13 μl of the oligo mixture containing RPA forward primer (exoPriFK1), reverse primer (exoPriRK2), probe (exoProK1), 2.5 μl of 14 mM Mg acetate and 5 μl of SwiftX supernatant were added into the lid of the tube (0.2 ml) with RPA lyophilized pellet. Designed primers construed from a conserved partial sequence of the *Leptospira interrogans* gene *lipL32*, (GenBank: AE016823.1, sequence 1666423 to 1666512) to detect pathogenic *Leptospira* precisely [15]. The primers and probe developed in a previous study (Ahmed *et al*. 2014) were used [15]. All the other reagents excluding rehydration buffer, magnesium acetate and primers/ probe were comprised as a freeze-dried pellet in the tube. Cap closed tube was centrifuged, vortexed and centrifuged and placed into the tube scanner. The test was performed on the portable, real-time fluorometer tube scanner Twista (TwistDx, Ltd., Cambridge, UK).

The machine was programmed at constant incubation period at 38°C followed by mixing and a spin down step at 230 s after the start of the incubation with a repeat incubation for 15 minutes with fluorescence detection. Total reaction time of the set up will be 15 minutes. The 6-carboxyfluorescein (FAM) fluorescence of RPA was generated with the cleavage of exonuclease III inducing detachment of quenched-Fluorophore at Tetrahydrofuran (THF) of the hybridized exo-probe. Real-time fluorescence detection of RPA amplicons and interpretation of the results was implemented using Twista Studio software, version 2.06.06 (TwistDx, Ltd.).

Each RPA run included nuclease-free distilled water (5 μl) as the negative control. Genomic DNA of *L*. *interrogans* serovar Icterhaemorrhagiae (culture supernatant) was extracted using QIAamp DNA mini kit (QIAGEN). The extracted DNA was quantified and used as the positive control in each reaction strip. A standard valid test was accepted with a defined exponential curve recorded above the threshold limit (250 mV) for the positive control of and a clear flat line under the threshold for the negative control. The tested samples were reported as positive with a valid exponential curve displayed above the. cut-off value of 300 mV [16].

### Laboratory confirmation by performing qPCR

*Leptospira* DNA was extracted from 200 μl of EDTA blood samples by using QIAamp DNA blood mini kit (QIAGEN GmbH, Hilden, Germany) according to the manufacturer’s instructions. Quantitative PCR was performed on the Swift Spectrum 48 Real Time Thermal Cycler (Esco Healthcare Pvt Ltd, Singapore) using the SYBR Green-I technology with primers (Genbank accession number AF115283) secYIVF (5’-GCGATTCAGTTTAATCCTGC-3’) and secYIV (5’GAGTTAGAGCTCAAATCG-3’) purchased from Integrated DNA Technologies, Inc., U.S.A. Primers are homologous to *S10-spc-α* locus of the *Leptospira interrogans* and amplify a 202 bp fragment between the locus positions 15744 and 15946. The reaction conditions and thermal conditions were optimized according to the previously established protocols of Ahmed *et al*. 2009 and Denipitiya *et al*. 2016 [11, 18]. The reference samples (*L*. *interrogans* serovar Icterohaemorrhagiae strain RGA and *L*. *biflexa* serovar Patoc strain Patoc I) were obtained from the National Leptospirosis Reference Laboratory at the Medical Research Institute (MRI) Sri Lanka [11]. Quantitative PCR reaction was performed using 5X HOT FIREPol® Evagreen® qPCR Mix Plus (ROX) master mixture (Solis Biodyne) in a total volume of 20 μL. The mixture contained FIREPol® Taq DNA polymerase and ultrapure dNTPs, 2.5 mM MgCl_2_ per reaction, EvaGreen® dye and ROX according to system requirements. Forward and reverse primers were added to a final concentration of 0.5 μM each followed by addition of 10 μl of extracted DNA (sample). Each PCR run included negative controls in which 10 μl of nuclease free distilled water was added instead of template DNA. DNA (10 μl) of *L*. *interrogans* serovar Icterhaemorrhagiae, strain RGA, Icterohaemorrhagiae serogroup was used as the positive control. The quantity of *Leptospira* genomic DNA of the positive control was ~3140 copies estimated using Quantus^TM^ Flurometer which was optimized with programmed settings for Promega QuantiFluor® dye systems [18].

Amplification process was carried out after initial denaturation at 95°C for 10 minutes. Thermal process of amplification for 40 cycles was carried out including steps of denaturation at 95°C for 15 s, annealing at 54°C for 30 s, extension at 72°C for 30 s, and with an extended final incubation at 72°C for 8 minutes. After cooling of 30°C for 1 minute, melting curve (Tm) analysis from 65°C-94°C with readings every 0.5°C was performed according to the manufacturer’s instructions. All test samples were repeated at least twice for maximum reproducibility. The cut off for the analysis was set at Threshold Cycle (Ct) value 35 and the approximate total time taken to run the entire program was about 2 hours. The melting temperatures ranging with a normalization region between 80°C and 85°C in the first active melting area were considered in evaluating positive samples. Data was analyzed using the spectrum PC software provided by the Swift Spectrum 48 fluorescence qPCR detection system [11, 19].

The qPCR and RPA were performed with each sample in triplicate. Samples were considered as leptospirosis positive if two or more replicates yielded positive reaction. Samples with single positive result was repeated in duplicate and was recorded positive if at least one more positive replicate was obtained. Further, all the tests were repeated twice [11].

### Microscopic agglutination test (MAT)

The Microscopic agglutination test was performed as the standard reference test in serodiagnosis of leptospirosis [9, 10, 20]. MAT was performed on all acute blood samples and convalescent blood samples collected 7 days after onset of the disease from the same patients. In Sri Lanka, MAT is performed according to standard protocol using common locally prevalent serovar panel (Table 1) at the National Leptospirosis Reference Laboratory, Medical Research Institute, Sri Lanka.

**Table 1:** Panel of *Leptospira* serogroups used as the standard MAT procedure for leptospirosis confirmation.

	*Leptospira* serogroups
1.	Patoc-MAT
2.	*L*.*interrogans* serovar australis, strain Balico in australis serogroup
3.	*L*.*interrogans* serovar bangkinang, strain bangkinang in 1 in Autumnalis serogroup
4.	*L*.*interrogans* serovar bataviae, strain Swart in Bataviae serogroup
5.	*L*.*interrogans* serovar bakeri, strain LT79 in Tarassovi serogroup
6.	*L*.*interrogans* serovar ratnapura, strain Wumalasena in Grippotyphosa serogroup
7.	*L*.*interrogans* serovar hardjo, strain Hardjoprajitno in Sejroe serogroup
8.	*L*.*interrogans* serovar icterhaemorrhagiae, strain RGA in Icterohaemorrhagiae serogroup
9.	*L*.*interrogans* serovar pyrogenes, strain Salinem in Pyrogenes serogroup
10.	*L*.*interrogans* serovar pomona, strain Pomona in Pomona serogroup
11.	*L*.*interrogans* serovar hebdomadis, strain Hebdomadis in Hebdomadis serogroup
12.	*L*.*interrogans* serovar cynopteri, strain 3522C in Cynopteri serogroup
13.	*L*.*interrogans* serovar patoc, strain Patoc 1 in Semaranga serogroup (Saprophytic Serogroup–genus
14.	*L*.*interrogans* serovar canicola, strain Hond Uterecht IV in canicola serogroup
15.	*L*.*interrogans* serovar poi, strain poi in Javanica serogroup
16.	*L*.*interrogans* serovar sarmin, strain Sarmin in Sarmin serogroup

Confirmation of the disease diagnosis of clinically suspected patients is based on MAT titer of ≥1:320 (cut-off recommended for Sri Lanka by the Reference Laboratory), a fourfold rise in titer or seroconversion from acute and convalescent sera [3, 17]. The serum samples were separated from the whole blood after centrifugation at 3,000 rpm for 15 minutes. The serum samples used within one month of collection was stored in the freezer -20°C for short term storage. Additionally, serum samples were kept in an ultra-low freezer -80°C for long-term storage. The blood serum separation and storage procedure was carried out within 2 hours of sample collection. The human serum samples were transported to the National Reference Laboratory for testing. Live antigen subculture and preparation procedure was established. Positive *Leptospira* serovar stock cultures were maintained in screw cap test tubes containing 5–6 ml of liquid EMJH media. The subcultures were made by inoculating 0.5 ml of the previous stock cultures from the panel of serovars/ strains into the new fresh culture media. The inoculated cultures were examined for the presence of viable *Leptospira* that were incubated at 30°C for a week. Cultures were examined under a dark-field microscope to confirm the presence of *Leptospira*. The procedure for the maintenance of the subcultures were performed every 7 days. The authenticity of the strains were routinely tested with the reference rabbit anti sera or monoclonal antibodies.

A 96 well flat bottom microtiter plate was filled with 50 μl Phosphate Buffered Saline (PBS) at pH 7.2. Then followed addition of another 50 μl of PBS and 10 μl of serum sample to the wells of column 2 making dilution of the serum. The serum is diluted by pipetting and adding to the next column of wells, thus final step discarded 50 μl. Dilution series of the serum samples were prepared accordingly from column 2 as 1:20, 1:40, 1:80, 1:160, 1:320, 1:640, 1:1,280, 1:2,560, 1:5,120, 1:10,240, and 1:20,480. The first column of plate contained antigen control loaded in 50 μl of PBS and 50 μl of live antigen strain making a final volume of 100 μl. The remaining rows were allocated for single serum sample and each column for single strain. Then the 50 μl of a specific live antigen was added to all the consecutive wells loaded with 50 μl of diluted sera. The plates were placed in a shaker for 5 minutes allowing optimal mixing after loading of samples and test strains, followed the step of incubation at 30°C for 2 hours. Finally, agglutination reaction was read by observing 5 μl drop from each well on a clean glass slide under dark field microscope (200X magnification). Reading done row wise where single serovar was tested at a time. Positive samples were chosen which gave 50% reduction free motile *Leptospira* comparatively with live antigen [21]. Disease confirmation of clinically suspected patient was based on the following criteria: MAT titer of ≥1:320 (cut-off), a fourfold rise in titer or seroconversion from acute and convalescent sera. In the present study, a detection of MAT titer of ≥1:320 was considered as the positive result.

### Data analysis

Computer-aided data bases for information on patient, laboratory clinical, laboratory testing of patient were processed in the Microsoft Excel spread sheets (Microsoft Office 2016). Index or reference test with missing/misplaced data will be excluded from the study. The diagnostic sensitivity, specificity, Positive Predictive Value (PPV), and Negative Predictive Value (NPV) of RPA-SwiftX and qPCR assays were calculated using standard equations in IBM SPSS Statistics, version 21 (IBM Corporation, NY, USA). The Kappa test was executed to evaluate agreement of the RPA and qPCR with standards. Interpretation of the results emphasize the agreement as: agreement equivalent to chance, fair agreement, moderate agreement, substantial agreement, and perfect agreement based on kappa values <0, 0.21–0.40, 0.41–0.60, 0.61–0.80, and 0.81–0.99 respectively [22].

Diagnostic performances of the RPA/SwiftX and qPCR assays were compared using McNemar’s exact test. Bayesian latent class model analysis for imperfect gold standards was performed using web-based application using R and WinBUGS programs on MICE (Modelling for Infectious Disease Centre, Mahidol-Oxford Research Unit) [23].

Guidelines of the Standards for Reporting of Diagnostic Accuracy Testing (STARD) were followed in this study [24].

## Results

Under the current study, 140 clinically suspected patients who fulfilled surveillance case definition of having leptospirosis were included for the analysis. The study group consisted of patients aged 12 to 77 years with the median age of 45 years. The affected patients were mainly males 84% (118/140). The retrospectively performed MAT became positive in 57.8% (81/140) of serum samples of suspected patients. The newly introduced RPA-SwiftX gave positive result in 42% (58/140) of patients and qPCR detected pathogenic *Leptospira* DNA in 45% (63/140) of suspected cases. These positive samples were quantified using qPCR. Of 81 positive MAT confirmed patients, 74%(60/81) and 65% (53/81) were positive with qPCR and RPA respectively.

Severity of the disease was investigated based on the criteria of the patient hospitalization and the referred to ICU (Intensive Care Unit) as the clinical symptoms and other laboratory data are diverse to analyze (S1 Table). All the selected patients were from the District of Gampaha, Sri Lanka, were hospitalized (100%) and 6.4% referred to ICU indicating the severity of the patient’s condition.

### Evaluation of the RPA and qPCR assay

All the assays (RPA-SwiftX, qPCR and MAT) were performed by investigators of the study. For the RPA assay and qPCR, blood samples collected during acute phase of the illness were used. The MAT was performed on convalescent serum samples collected after a minimum of 7days following onset of clinical disease. The comparison results of index tests (qPCR and RPA-SwiftX with the results of the reference standard MAT were used for the analysis (S2 Table).

**Fig 1:**
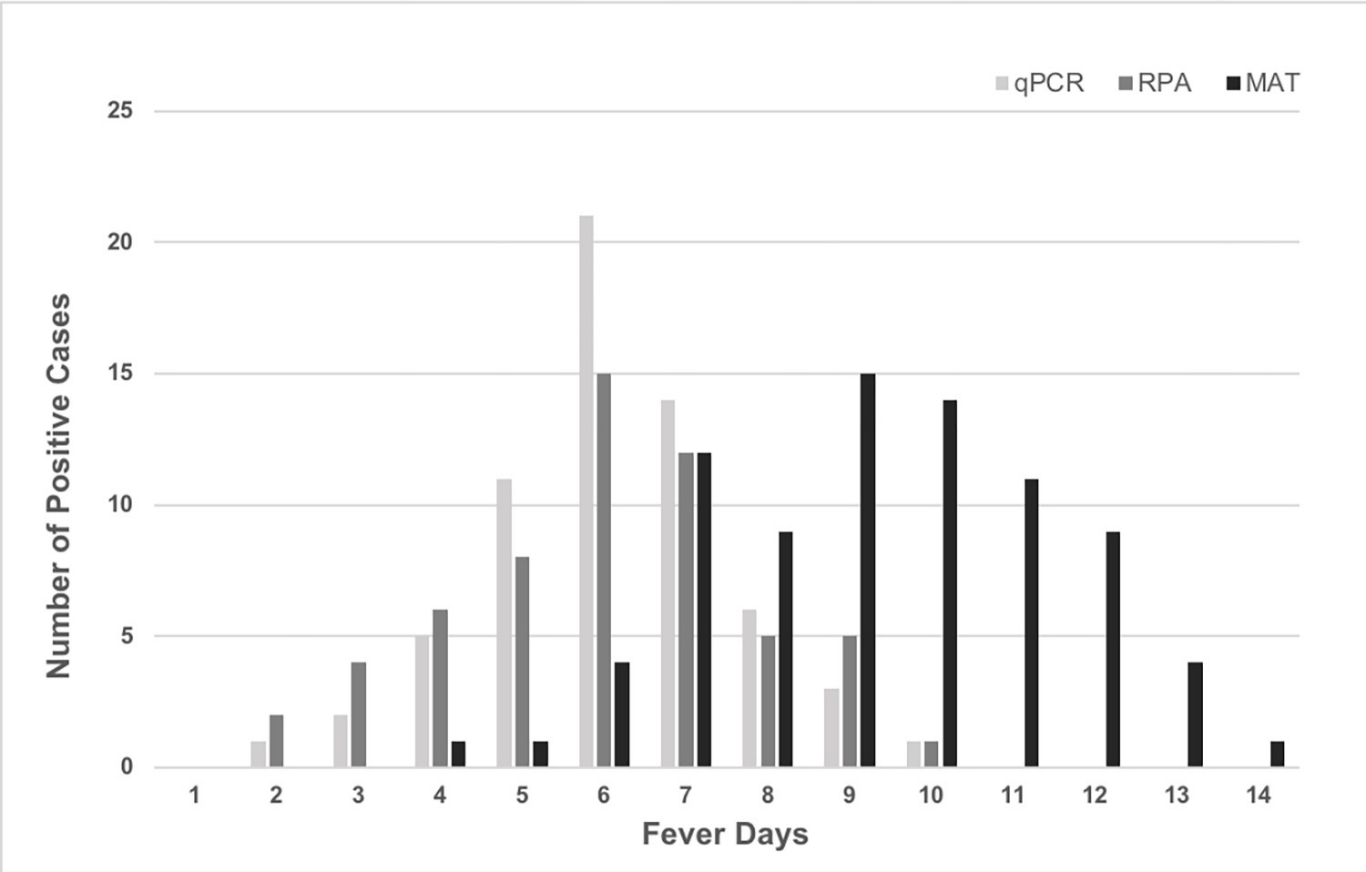
Evaluation of leptospirosis confirmed incidence based on molecular (qPCR and RPA) and serological diagnostic method (MAT) against the fever days at the sample collection post initial consultation. The bars show the number of positive cases of each test of qPCR, RPA, and MAT in the days post-onset of the disease of the patients at the sample collection.

Results indicate that RPA and qPCR assays tested positive in 81% (98/121) of the total positive cases from whom blood was collected within the first 7 days of fever (Fig 1). According to the results, the proportion of positive incidence with the nucleic acid amplification assays drastically decreased to 19% of total prevalence after 7 days of illness. Furthermore, 85% and 77% patients admitted before 7 days post-onset of the disease (DPO) tested positive of total positives of qPCR and RPA, respectively. MAT as a serological assay which indicates antibody formation was positive in 78% of the samples collected 7days after onset of illness. However, early seroconversion was noted with 22% of the tested samples. According to this study, the highest number of positive cases were determined in the 6^th^ day of onset of the disease.

**Fig 2:**
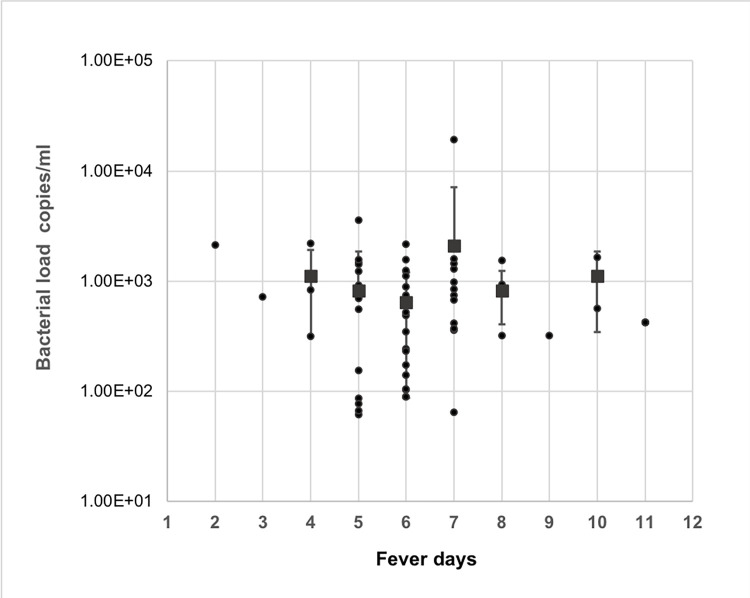
Comparison of quantified bacterial load of qPCR positive cases for pathogenic *Leptospira* DNA against the admission fever day. Each dot represents the bacterial copies of individual patients. Error bars indicate standard deviations. The Lower Limit of Detection (LOD) of qPCR was approximately 210 genome copies based on the standard curve constructed from serial dilutions of genomic DNA.

*Leptospira* DNA was detected in patients from fever day 2 to day 11 with a bacterial load ranging from 62–19,400 copies/ml (Fig 2). Mean bacterial loads signified for the fever days 4,5,6,7,8 and 10 were 1,113, 824, 642, 2,115, 824 and 1,109 copies/ml respectively. According to the results, the highest mean bacterial load 2,115 copies/ml observed in the 7^th^ DPO.

The performance of the RPA-SwiftX and qPCR assays in comparison to the MAT assay is summarized in Table 2 assessing diagnostic sensitivity, specificity, PPV and NPV of the assays. Kappa (κ) values of 0.66 and 0.54 were obtained when the qPCR and RPA-SwiftX was compared with the MAT assay respectively indicating the substantial agreement of qPCR and moderate agreement of RPA-SwiftX with MAT. Results suggest that there is no significant difference between two assays qPCR and RPA- SwiftX (P = 0.40) (Table 2). No significant adverse effects occurred during results interpretation.

**Table 2:** Diagnostic performance of the RT-RPA that of the q-PCR assay in serum samples (n = 140) from patients with clinically suspected leptospirosis.

Assay and Results	qPCR					
	Positive (n = 63)	Negative (n = 77)	Sensitivity (%[95% CI])	Specificity (% [95% CI])	PPV (%[95% CI])	NPV (%[95% CI])
**RPA- SwiftX**						
Positive (n = 58)	49	9	77(70–84)	88(82–93)	84(78–90)	82(76–89)
Negative(n = 82)	14	68				

Diagnostic sensitivities of qPCR and RPA-SwiftX according to the comparison results with gold standard MAT were 79% and 70% correspondingly (Table 3). Conversely, Specificity and PPV of qPCR (≥90%) and RPA-SwiftX (≥87%) was significant. Remarkably, Bayesian Latent Class Model estimates of unbiased sensitivities of qPCR and MAT were comparatively higher than those estimated by assuming MAT as gold standard. Estimated unbiased diagnostic specificities and PPV of qPCR (≥99%) and RPA-SwiftX (≥96%) were considerably higher to those derived from conventional analyses.

**Table 3:** Diagnostic parameters (Sensitivity, specificity, PPV and NPV) estimated by using MAT as the reference standard and Bayesian latent class model (LCM).

Parameters	MAT was assumed as a perfect gold standard (%)*	Bayesian latent class model (%) **
**MAT**		
Sensitivity	100	90.9 (81.9–96.7)
Specificity	100	85.4 (73.5–95.3)
PPV	100	87.8 (76.6–96.4)
NPV	100	89.1 (78.0–96.0)
**q PCR**		
Sensitivity	79.1 (67.1–87.7)	89.6 (78.0–97.5)
Specificity	90.7 (78.9–96.5)	99.5 (94.7–100)
PPV	91.4 (80.3–96.8)	99.5 (94.9–100)
NPV	77.8 (65.2–86.9)	89.2 (75.9–97.4)
**RPA- SwiftX**		
Sensitivity	70.1 (57.6–80.4)	80.6 (68.6–89.3)
Specificity	87.0 (74.5–94.2)	96.8 (87.2–100)
PPV	87.0 (74.5–94.2)	96.6 (86.4–100)
NPV	70.1 (57.6–80.4)	81.2 (68.6–90.1)

* Gold standard model assumed that MAT is perfect. Accordingly, all patients with gold standard test positive are confirmed leptospirosis and negative are non-diseased. Estimated means statistically performed with 95% confidence interval.

** Further Bayesian latent class model presumed all tests are imperfectly evaluated. Values shown are estimated median with 95% confidence interval.

## Discussion

Leptospirosis is a zoonotic infectious disease associated with high morbidity and mortality. It occurs worldwide with a higher incidence in the tropics and subtropics compared to temperate regions. The incidences of leptospirosis range from 0.1-1/100,000/year in temperate climates, and 10–100/100,000/year in the tropic. The reported incidence significantly changes with frequently occurring outbreaks in any setting. A high level of endemicity is reported in the Caribbean, Central and South America, Southeast Asia, and Oceania [25]. Patients infected with *Leptospira* present mild to severe life-threatening disease [6]. The clinical diagnosis of the disease is challenging even in disease-prevalent settings due to nonspecific and highly variable clinical manifestations [6, 26]. Prompt diagnosis and timely commencement of antibiotics with other supportive care is essential for favorable outcome. Hence the disease prevalent low resource countries require a highly sensitive, low-cost, specific test with a short turnaround time for active cases detection during the acute phase of illness. Furthermore, validation of an assay having a potential to use as a point of care diagnostic platform will provide additional support for active case detection, epidemiological surveillance, and disease control activities [16, 27]. The present study evaluated the isothermal amplification method RPA with a qPCR assay for the acute phase detection of leptospirosis cases. Both assays were further compared with MAT, the gold standard test for the laboratory confirmation of leptospirosis.

A variety of diagnostic tests have been developed and are in use for the acute phase diagnosis of leptospirosis. However, the availability of such facilities is limited especially in disease-prevalent low-resource settings. Direct observation of leptospires by darkfield microscopy is unreliable and not recommended for routine clinical use. The reported sensitivity of darkfield microscopy is approximately 10^7^/L. Moreover, the test needs sophisticated laboratory facilities and skilled human resources [12]. Leptospires are not stained by conventional Gram staining. The stains used to increase the sensitivity of direct microscopic examinations include immunofluorescence, immunoperoxidase, silver staining, Warthin-Starry staining, immunohistochemistry, and in situ hybridization [13, 28]. However, all of these carry a high risk of false-positive and false-negative results, especially on inexperienced hands. Although the organism can be cultured from clinical specimens (blood, CSF, and dialysate) collected during the acute phase of the illness, isolation of leptospires can take up to months and does not contribute to early clinical diagnosis [29]. The urine samples should be collected for cultures during the second week of illness. Different antigen detection tests had been developed but none had given promising results to use in clinical practice [13].

In recent years, several real-time PCR assays have been developed for acute phase diagnosis [11]. PCR detects DNA in blood in the first 5 to 10 days after the onset of the disease and up to the 15^th^ day. The bacterial load in serum/blood ranges from 10^5^ to 10^9^ leptospires/L. The published results on PCR-based molecular diagnostic tests show a variation in the clinical specimen used. Such studies used serum, plasma, whole blood, and buffy coat with mixed results [13]. In the present study, the testing was done on plasma and the study showed a 79% sensitivity and a specificity of 90% with qPCR compared to MAT, the gold standard. However, prolonged turnaround time, need of laboratory facilities and high running cost has made the technique infrequently available in disease prevalent low resource settings.

The usual practice is to clinically suspect leptospirosis based on case definitions or diagnostic criteria with possible risk exposures [30]. Such patients are prescribed antibiotics together with supportive care for organ dysfunction. In most settings, convalescent serum samples collected after 7 days of disease onset from patients are sent to the reference laboratories for MAT. This is for retrospective confirmation of clinical diagnosis. Positive MAT results are likely be confirmed from Day 10 to 12 after the onset of illness, sometimes later if appropriate antibiotics have been prescribed. The reported sensitivity of MAT is 41% during the 1^st^ week, 82% during the 2^nd^ to 4^th^ week, and 96% beyond the 4^th^ week of illness [13]. In the present study, 140 clinically suspected patients, who were clinically managed as leptospirosis cases, 81 gave positive results with MAT. The cutoff value for a single serum sample depends on the seroprevalence and different values have been used in different settings. In the present study, the value used was ≥1:320. Moreover, the MAT allows the identification of the possible serogroups involved in this cohort of the population (for more information see S3 Table of supplementary materials). Enzyme-Linked Immunosorbent Assays (ELISAs) are used in some centers for serological confirmation. The main limitation of serology is that antibodies are lacking at the acute phase of the disease.

Rapid diagnostic tests are increasingly used for the early detection of infectious diseases due to their short turnaround time, easy handling, and no need of trained human resources. However, interobserver variability in reading and interpretation of the endpoints may provide inconsistent results. The majority of RDTs are primarily IgM detection assays. Patients with leptospirosis usually mount an IgM response during the second week of illness. Therefore, such tests cannot be used in acute phase diagnosis. Eight rapid tests (IHA, 2 IgM dipstick assay; indirect fluorescent antibody, 3 ELISA IgM, LA) have been evaluated in Hawaii and have concluded that all tests were insensitive for diagnosis (test sensitivity was particularly low <25%) within the first week of the disease [31].

Isothermal techniques have recently been developed for the diagnosis of leptospirosis comprising nucleic acid sequence–based amplification (NASBA), loop-mediated isothermal amplification (LAMP), helicase-dependent amplification, rolling circle amplification, strand displacement amplification, and recombinase polymerase amplification (RPA) [12, 15, 32, 33]. Isothermal amplification is considered as an alternative to PCR-based method. It is advantageous since a simple heating device is needed to maintain a constant temperature instead of complex thermocyclers used in PCR. Technology can be implemented in resource-poor settings at the point of need in epidemics. However, the use of PCR has dominated its use in diagnostics due to advanced technology, although the isothermal process is more beneficial in use as a simple and affordable approach. The development of diagnostics that can be performed with limited laboratory equipment is important since leptospirosis affects mainly people in resource-poor settings [34]. This requirement is fulfilled with novel isothermal applications like LAMP and RPA.

LAMP has enabled direct amplification of clinical specimens without an additional purification step. Rapid detection of pathogenic *Leptospira* spp targeting the *lipL41*, *lipL32* or rrs genes were developed with moderate diagnostic specificity and the limit of detection 2–100 leptospires per reaction [12, 32, 33]. However, some studies emphasize that LAMP cannot compete with the diagnostic sensitivity and specificity of rtPCR [12, 35, 36].

Recombinase polymerase amplification (RPA) has been developed as a novel isothermal amplification technique. Several advantages of RPA over other amplification methods emphasize its potential for implementation in a basic laboratory setting in the field. The test was performed at an ambient temperature using a recombinase enzyme, single-stranded DNA binding proteins, and homologous oligonucleotides. In RPA reaction, the reaction is performed at a moderate and constant temperature (37–39°C) and sequence-specific priming of DNA polymerase reaction has been endorsed without denaturation of template DNA. The application is implemented in a point of care situation, whereas the technique facilitates real-time readout or endpoint “sandwich assays”, such as lateral flow strips. Studies evaluated an RPA application for the detection of pathogenic *Leptospira* using a TwistAmp Exo probe detection system in collaboration with real-time readout [15, 37].

The need for highly sensitive and specific rapid diagnostics at the time of admission has led to the development of numerous molecular-based isothermal assays. Moreover, the availability of adequate laboratory facilities provides strong support for disease control through surveillance. Although adequate laboratory support exists in developed countries, they are often lacking in leptospirosis endemic developing countries. Hence, leptospirosis is often underestimated in such settings. The newly introduced RPA assay with the potential for use in point of care diagnosis during the acute phase of disease showed 70% sensitivity and 87% specificity compared to gold standard MAT. Moreover, the RPA technique gives results in 15 minutes. The combined RPA-SwiftX method under field conditions took only 35 minutes compared to the qPCR which required 3 hours to provide the results. The diagnostic sensitivities of qPCR and RPA were 79% and 70% with a significant specificity and a positive predictive value of qPCR (≥90%) and RPA-SwiftX (≥87%). Molecular assays such as qPCR, RPA, and LAMP detect pathogenic *Leptospira* with high diagnostic specificities [11, 12, 15, 32, 33]. However, sample transportation, PCR inhibitors, and partial digestion of the serum samples in the DNA extraction would affect the sensitivity of RPA. Moreover, the lower diagnostic sensitivity of the RPA-SwiftX compared to qPCR may be due to the implementation of rapid extraction method (SwiftX) as crude DNA extracted can inhibit the amplification process. Nonetheless, RPA-SwiftX showed 77% sensitivity compared to qPCR, suggesting that there was no significant difference between the two assays (P = 0.40). The study has evaluated molecular based assays, RPA and qPCR to determine disease prediction in the early acute phase of the illness. However, the time interval between the molecular based assays (qPCR and RPA) and MAT as the serology might have an adverse consequence on the test results due to the changes occurred in the target conditions. The diagnostic sensitivity of the test might be underestimated due to the prolonged performance of the reference test. The patients could be suggestively not reflect the early acute phase of the illness since they misinterpret the fever days at the admission and relatively late hospitalization. In such a situation, a higher incidence of false negative results might occur underrating the diagnostic sensitivity of the index test.

The use of the RPA- SwiftX assay has many advantages at the point-of-need. Affordable price of approximately 10 USD per reaction and availability at the field level performance would be advantageous compared to sophisticated and expensive qPCR practice.

## Conclusions

The sensitivity of any molecular-based assays is strongly affected by DNA extraction method, sample transportation, and storage. RPA-SwiftX assay shows the potential of using it as a point of care diagnostic test for leptospirosis. Findings suggest that the sample collection should be considered together with the patient’s DPO to get the most accurate laboratory diagnosis. MAT accurately detects late acute phase infections but takes several days to deliver results. The assays RPA-SwiftX and qPCR can detect acute phase infections, with acceptable diagnostic sensitivity for clinical use.

## Supporting information

S1 Fig Flow diagram of index patients included in the study showing the positive results of index and reference tests. (TIFF)

S1 Table Information of index patients. Hospitalization and ICU (intensive care unit); N, no; Y, yes; U, Test results; -, negative; +,positive.
(PDF)

S2 Table The comparison results of index tests (qPCR and RPA-SwiftX with the results of the reference standard MAT. (PDF)

S3 Table Identification of pathogenic *Leptospira* in human using serogroup specific MAT. (PDF)
