# Supplementary figures and images for: Detection of pathogenic Leptospira with rapid extraction followed by recombinase polymerase amplification (RPA) and quantitative polymerase chain reaction (qPCR) assay-A comprehensive study from Sri Lanka
Source: PLoS One. 2024 Mar 15;19(3):e0295287. doi: 10.1371/journal.pone.0295287 (PMC10942058; doi:10.1371/journal.pone.0295287)

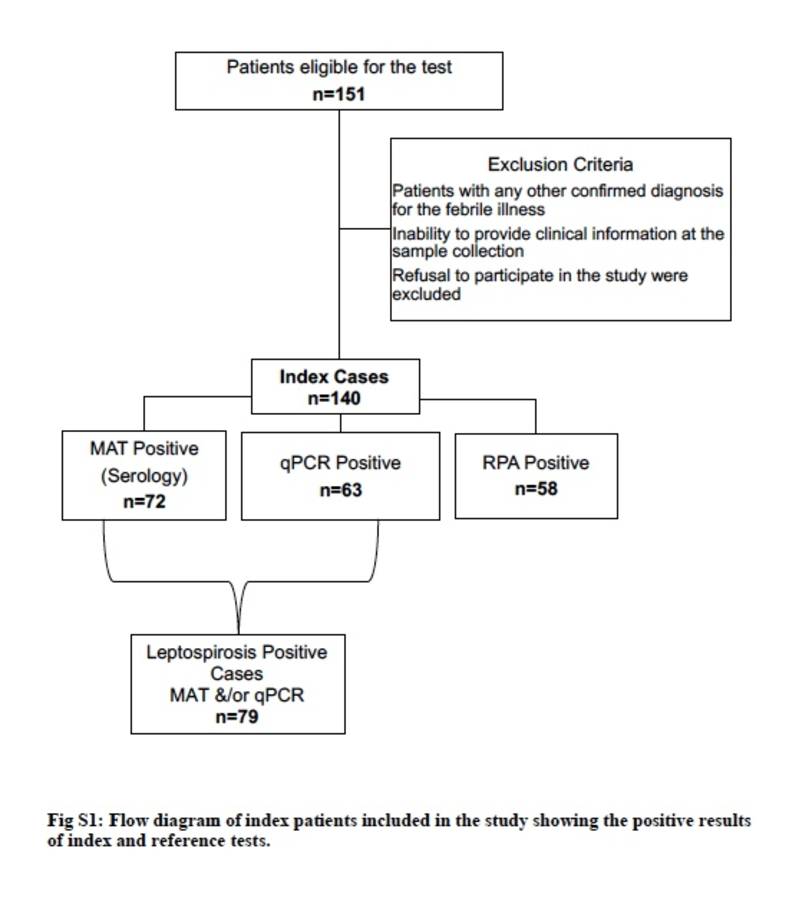

Supplement: S1 Fig — (TIFF) [file pone.0295287.s001.tiff]
