# Supplementary material for: Detection of pathogenic Leptospira with rapid extraction followed by recombinase polymerase amplification (RPA) and quantitative polymerase chain reaction (qPCR) assay-A comprehensive study from Sri Lanka
Source: PLoS One. 2024 Mar 15;19(3):e0295287. doi: 10.1371/journal.pone.0295287 (PMC10942058; doi:10.1371/journal.pone.0295287)
Supplement: S1 Table — Hospitalization and ICU (intensive care unit); N, no; Y, yes; U, Test results; -, negative; +,positive. (PDF) [file pone.0295287.s002.pdf]

Table S1. Information of index patients.

Index Patient	Lab ID	Age	Fever Days *	Gender	Hospitalization	ICU	MAT	PCR	RPA
1	R001	37	7	M	Y	Y	+	+	+
2	R002	58	6	M	Y	N	+	+	+
3	W001	50	5	M	Y	Y	-	-	-
4	W002	43	4	M	Y	N	-	-	-
5	W003	50	5	M	Y	N	-	-	-
6	W005	50	6	M	Y	N	+	+	-
7	W006	39	6	M	Y	N	+	+	-
8	W007	22	2	M	Y	N	-	-	-
9	W008	39	6	M	Y	N	+	+	-
10	G001	15	6	M	Y	N	-	-	-
11	R010	45	7	M	Y	N	+	+	-
12	N002	22	6	M	Y	N	-	-	-
13	N003	50	5	M	Y	N	-	-	-
14	N008	31	4	M	Y	N	-	-	-
15	R005	60	6	M	Y	N	+	+	+
16	R006	50	5	M	Y	N	+	+	+
17	R008	62	2	M	Y	Y	-	+	+
18	W009	48	6	M	Y	N	+	+	+
19	W010	19	7	M	Y	N	+	+	+
20	W011	69	4	M	Y	N	+	+	+
21	W012	35	7	M	Y	N	+	+	-
22	R011	76	6	M	Y	N	+	+	+
23	R012	18	**5**	M	Y	N	+	+	+
24	W015	53	3	M	Y	N	+	+	-
25	W016	64	5	M	Y	N	+	+	-
26	W017	54	6	M	Y	N	+	+	-
27	W018	52	6	M	Y	N	+	+	-
28	W019	37	5	M	Y	N	+	+	-
29	W020	39	7	M	Y	N	+	+	-
30	G007	53	7	F	Y	N	+	+	+
31	W021	35	2	M	Y	N	-	-	+
32	W022	48	6	M	Y	N	+	+	+
33	W023	51	7	F	Y	N	+	+	+
34	W024	56	7	F	Y	Y	+	+	+
35	W025	43	7	M	Y	N	+	+	+
36	W026	41	5	M	Y	N	-	-	-
37	H001	29	6	F	Y	N	+	-	-
38	H002	19	5	M	Y	N	+	-	-
39	H004	45	10	M	Y	N	+	-	-
40	H005	13	6	F	Y	N	+	+	-
41	H006	47	6	M	Y	N	+	-	-
42	H007	37	3	M	Y	N	+	-	+
43	H008	30	4	M	Y	N	-	-	-
44	H009	80	8	F	Y	N	+	+	-
45	H010	37	6	M	Y	N	+	+	+
46	H011	20	3	M	Y	N	+	-	-
47	H012	35	5	F	Y	N	-	-	-
48	H013	43	7	M	Y	N	+	+	+
49	W026	35	7	M	Y	N	-	-	-
50	W027 1	76	7	M	Y	N	-	-	-
51	W027	30	7	M	Y	N	+	-	+
52	W028	48	4	M	Y	N	+	-	+
53	H014	74	5	F	Y	N	-	-	-
54	H015	50	6	M	Y	N	+	+	+

55	H016	20	5	M	Y	N	+	+	+
56	H017	53	7	M	Y	N	+	-	+
57	H018	52	8	F	Y	N	+	-	-
58	H019	53	9	M	Y	N	+	-	-
59	H020	65	3	M	Y	N	+	-	+
60	H021	40	7	M	Y	N	+	-	-
61	W029	49	6	M	Y	N	+	+	+
62	W030	68	7	M	Y	N	-	-	-
63	W031	52	5	F	Y	N	+	+	-
64	W032	38	9	F	Y	N	+	-	+
65	W033	23	7	M	Y	N	+	-	-
66	W034	43	5	M	Y	N	-	-	-
67	W035	40	6	M	Y	N	+	+	+
68	H022	64	7	M	Y	N	-	-	-
69	H023	38	9	M	Y	N	+	-	-
70	H024	76	8	M	Y	N	+	-	-
71	H025	42	6	M	Y	N	+	-	-
72	H026	40	5	M	Y	N	+	+	+
73	H027	47	7	M	Y	N	-	-	-
74	H028	49	7	M	Y	N	-	-	-
75	H029	54	5	M	Y	N	-	-	-
76	H030	62	4	M	Y	N	-	-	-
77	H031	52	6	F	Y	N	-	-	-
78	W036	38	7	M	Y	N	-	-	-
79	W037	24	3	M	Y	N	+	+	+
80	H035	64	5	M	Y	N	+	+	+
81	H036	46	5	M	Y	N	+	+	+
82	H037	27	6	M	Y	N	+	-	-
83	H038	33	7	M	Y	N	+	+	-
84	H039	26	3	M	Y	N	+	-	+
85	W038	21	6	M	Y	N	-	-	-
86	W039	47	9	M	Y	N	+	-	+
87	W040	45	7	M	Y	N	-	-	-
88	H041	60	8	M	Y	Y	+	+	+
89	H42	59	7	M	Y	N	+	+	+
90	H43	45	8	M	Y	N	+	+	+
91	W44	12	8	M	Y	N	+	+	+
92	W45	46	6	M	Y	N	-	-	-
93	W046	45	6	M	Y	N	+	+	+
94	W047	29	4	M	Y	N	-	-	-
95	N010	27	5	M	Y	N	+	+	+
96	G005	65	6	M	Y	N	+	+	+
97	G006	42	5	M	Y	N	+	+	+
98	P1	36	4	M	Y	N	+	+	+
99	P2	49	9	M	Y	N	+	+	+
100	P3	34	6	M	Y	N	+	+	+
101	P4	16	6	M	Y	N	-	-	-
102	P5	51	7	M	Y	N	-	-	-
103	P6	46	9	M	Y	N	+	+	+
104	P7	28	7	F	Y	N	+	+	+
105	P8	18	6	M	Y	N	+	+	+
106	P9	41	6	M	Y	N	+	+	+
107	P10	34	10	M	Y	N	+	+	+
108	P11	57	3	M	Y	N	-	-	-
109	P12	45	9	M	Y	N	+	+	+
110	P13	76	8	M	Y	N	+	+	+
111	P16	62	7	M	Y	N	-	-	-
112	P17	54	7	M	Y	N	+	+	+

113	P18	50	6	M	Y	N	+	+	+
114	P19	75	4	M	Y	N	-	-	-
115	P20	17	8	M	Y	N	+	+	+
116	K1	42	4	M	Y	Y	-	+	+
117	K2	57	6	F	Y	Y	-	-	-
118	K3	46	4	M	Y	Y	-	+	+
119	K4	34	4	M	Y	Y	-	+	+
120	N015	50	7	M	Y	N	-	-	-
121	N009	42	5	M	Y	N	-	-	-
122	G002	50	3	F	Y	N	-	-	-
123	P21	62	4	F	Y	N	-	-	-
124	P22	27	5	M	Y	N	-	-	-
125	P23	56	7	F	Y	N	-	-	-
126	P24	41	7	M	Y	N	-	-	-
127	P25	38	5	M	Y	N	-	-	-
128	P26	43	6	F	Y	N	-	-	-
129	P27	50	3	M	Y	N	-	-	-
130	P28	42	4	F	Y	N	-	-	-
131	P29	33	6	M	Y	N	-	-	-
132	P30	37	7	M	Y	N	-	-	-
133	P31	60	5	F	Y	N	-	-	-
134	P32	55	4	M	Y	N	-	-	-
135	P33	29	5	M	Y	N	-	-	-
136	P34	51	2	F	Y	N	-	-	-
137	P35	38	8	M	Y	N	-	-	-
138	P36	40	2	M	Y	N	-	-	-
139	P37	35	7	F	Y	N	-	-	-
140	P38	62	1	M	Y	N	-	-	-

* Fever day at the first sample collection
Hospitalization and ICU (intensive care unit); N, no; Y, yes; U, Test results; -,negative; +,positive
