# Supplementary material for: Detection of pathogenic Leptospira with rapid extraction followed by recombinase polymerase amplification (RPA) and quantitative polymerase chain reaction (qPCR) assay-A comprehensive study from Sri Lanka
Source: PLoS One. 2024 Mar 15;19(3):e0295287. doi: 10.1371/journal.pone.0295287 (PMC10942058; doi:10.1371/journal.pone.0295287)
Supplement: S2 Table — (PDF) [file pone.0295287.s003.pdf]

S2 Table : The comparison results of index tests (qPCR and RPA-SwiftX with the results of the reference standard MAT.

Diagnostic Test	Reference Test	TP	FP	TN	FN
qPCR	MAT	60	3	56	21
RPA-SwiftX	MAT	53	5	54	28

*TP, true positive; FP, false positive; TN, true negative; FN, false negative
