# Supplementary material for: Detection of pathogenic Leptospira with rapid extraction followed by recombinase polymerase amplification (RPA) and quantitative polymerase chain reaction (qPCR) assay-A comprehensive study from Sri Lanka
Source: PLoS One. 2024 Mar 15;19(3):e0295287. doi: 10.1371/journal.pone.0295287 (PMC10942058; doi:10.1371/journal.pone.0295287)
Supplement: S3 Table — (PDF) [file pone.0295287.s004.pdf]

S3 Table. Identification of pathogenic *Leptospira* in human using serogroup specific MAT

	**Positive MAT**	**Percentage %**
Patoc-MAT	6	6.5
L.interrogans serovar australis , strain Balico in australis serogroup	**15**	**16.3**
L.interrogans serovar bangkinang , strain bangkinang in 1 in Autumnalis serogroup	7	7.6
L.interrogans serovar bataviae , strain Swart in Bataviae serogroup	1	1.1
L.interrogans serovar bakeri , strain LT79 in Tarassovi serogroup	**82**	**89.1**
L.interrogans serovar ratnapura , strain Wumalasena in Grippotyphosa serogroup	3	3.3
L.interrogans serovar hardjo , strain Hardjoprajitno in Sejroe serogroup	1	1.1
L.interrogans serovar icterhaemorrhagiae , strain RGA in Icterohaemorrhagiae serogroup	1	1.1
L.interrogans serovar pyrogenes, strain Salinem in Pyrogenes serogroup	9	9.8
L.interrogans serovar pomona , strain Pomona in Pomona serogroup	**12**	**13.0**
L.interrogans serovar hebdomadis , strain Hebdomadis in Hebdomadis serogroup	5	5.4
L.interrogans serovar cynopteri, strain 3522C in Cynopteri serogroup	8	8.7
L.interrogans serovar patoc , strain Patoc 1 in Semaranga serogroup	0	0.0
L.interrogans serovar canicola , strain Hond Uterecht IV in canicola serogroup	6	6.5
L.interrogans serovar poi , strain poi in Javanica serogroup	2	2.2
L.interrogans serovar sarmin , strain Sarmin in Sarmin serogroup	3	3.3
